# Noise cancellation using total variation for copy number variation detection

**DOI:** 10.1186/s12859-018-2332-x

**Published:** 2018-10-22

**Authors:** Fatima Zare, Abdelrahman Hosny, Sheida Nabavi

**Affiliations:** 1 Computer Science and Engineering Department, University of Connecticut, Storrs, CT USA; 2 Computer Science and Engineering Department and Institute for Systems Genomics, University of Connecticut, Storrs, CT USA

**Keywords:** Next generation sequencing, Copy number variation, Signal processing, Total variation, Taut string, Denoising

## Abstract

**Background:**

Due to recent advances in sequencing technologies, sequence-based analysis has been widely applied to detecting copy number variations (CNVs). There are several techniques for identifying CNVs using next generation sequencing (NGS) data, however methods employing depth of coverage or read depth (RD) have recently become a main technique to identify CNVs. The main assumption of the RD-based CNV detection methods is that the readcount value at a specific genomic location is correlated with the copy number at that location. However, readcount data’s noise and biases distort the association between the readcounts and copy numbers. For more accurate CNV identification, these biases and noise need to be mitigated. In this work, to detect CNVs more precisely and efficiently we propose a novel denoising method based on the total variation approach and the Taut String algorithm.

**Results:**

To investigate the performance of the proposed denoising method, we computed sensitivities, false discovery rates and specificities of CNV detection when employing denoising, using both simulated and real data. We also compared the performance of the proposed denoising method, Taut String, with that of the commonly used approaches such as moving average (MA) and discrete wavelet transforms (DWT) in terms of sensitivity of detecting true CNVs and time complexity. The results show that Taut String works better than DWT and MA and has a better power to identify very narrow CNVs. The ability of Taut String denoising in preserving CNV segments’ breakpoints and narrow CNVs increases the detection accuracy of segmentation algorithms, resulting in higher sensitivities and lower false discovery rates.

**Conclusions:**

In this study, we proposed a new denoising method for sequence-based CNV detection based on a signal processing technique. Existing CNV detection algorithms identify many false CNV segments and fail in detecting short CNV segments due to noise and biases. Employing an effective and efficient denoising method can significantly enhance the detection accuracy of the CNV segmentation algorithms. Advanced denoising methods from the signal processing field can be employed to implement such algorithms. We showed that non-linear denoising methods that consider sparsity and piecewise constant characteristics of CNV data result in better performance in CNV detection.

## Background

Understating the inherited basis of genomic variations and their contribution to phenotypes is the major goal of genomics. One of the main types of genomic variation is copy number variation (CNV), defined as a phenomenon in which sections of a genome, ranging from a few hundred base pairs to a few mega base pairs, are repeated or deleted [1, 2]. It is observed that 4.8–9.5% of the genome contributes to CNV and they affect more nucleotides per genome compared to single nucleotide polymorphism (SNP) [3]. CNVs can change gene dosage, create new genes, reshape gene structures, and modify gene expression regulatory elements [4, 5], and as a result they can significantly influence gene expression and phenotypic variation [6]. CNVs, particularly exon rearranging and gene duplication, can be a major procedure driving gene and genome evolution [7]. CNVs are associated with genetic disease susceptibility [7, 8], evolution and normal phenotypic variation. Recently several studies have indicated that there is a relationship between CNVs and many diseases including cancer [9]. CNV assessment is also important in functional genomic studies since not considering CNVs can result in misinterpretation of gene expression, methylation or chromatin immunoprecipitation data [10, 11]. The power to discover a relationship between genomic variation and phenotype is limited by the sensitivity, accuracy and comprehensibility of genomic variation identification methods. As a result, precise and efficient detection of CNVs, and accessible CNV detection software tools are very important in the advancement of genomics.

For studying CNVs, using array-based technologies has been a popular approach since late 1990s due to their reasonable cost and relatively high resolution. [12]. With the arrival of next generation sequencing (NGS) technologies [13] in the late 2000s and early 2010s; and because of limitations of array-based technology associated with hybridization and resolution, sequence-based CNV detection has become a more popular approach to detect CNVs with higher accuracy and resolution [14]. Consequently, several computational tools have been developed to identify CNVs using NGS data. However, accurate detection of CNVs from NGS data remains challenging [15] for a variety of reasons, including the big data nature of the NGS data, short-read lengths, sequence-specific biases, library preparation biases, and high level of noise. Comparative analyses of the performance of the CNV identification tools show that the tools’ false positive rates are high and agreements across the tools is low [16–18].

In general, there are four major methods to detect CNVs from NGS data: 1) read depth, 2) paired-end reads, 3) split reads, and 4) assembly [19–21]. Compared to other methods, RD-based methods can identify the exact number of CNVs, as the paired-end and split read approaches can only detect the position of the potential CNVs and not the copy numbers. Furthermore, RD-based approaches can work better on large sized CNVs, which are hard to detect by the paired-end and split read approaches [22]. Assembly-based methods are used less often in CNV detection because they are computationally very demanding. Furthermore, eukaryotic genomes contain a remarkable segmental duplication that render poor performance of assembly-based methods in these complex regions. Another problem with assembly-based approaches is that they fail to handle haplotype sequences and as a result only homozygous structural variations can be identified [23]. With availability of high-coverage NGS data and because of the above reasons, RD-based approaches have recently become a main method to detect CNVs, particularly for targeted sequencing data such as whole exome sequencing (WES) data. Mostly, in the RD-based approach, a non-overlapping sliding window is utilized to measure the number of reads that are aligned to a genomic region overlapped with the window. It is hypothesized that the number of short sequences that align to a position in the genome (readcount) is proportional to the copy number at that position. The readcount values, are used to detect CNV regions using segmentation methods [24–34]. However, existence of biases and noise distorts the correlation between copy numbers and the readcount values, which reduces the RD-based methods’ ability to detect CNVs accurately. Hence, a robust CNV detection method requires elimination of biases and noise from data before detecting CNVs. In general RD-based approaches include two main steps: 1) preprocessing, and 2) segmentation. In the preprocessing step, readcount data are generated, low quality read counts are removed, and readcount data are normalized to reduce bias. Even though readcount data is very noisy, most of the CNV detection methods do not employ denoising as part of their preprocessing step.

There are several sources for biases and noise in NGS data such as GC bias, mappability bias, sample contamination, sequencing noise, and experimental noise. GC content has been found to affect read coverage on most sequencing platforms and varies significantly along the genome [35–37]. Due to biochemical differences in the sequenced DNA, sequencing technologies act differently on sequences with different GC content [38]. It has been shown that segments of genome with low or high GC content have low readcounts compared to other segments. As a result, there is a unimodal correlation between readcounts and G and C bases in the genome [39–42]. Although the global structure of the distribution of readcounts with respect to the GC content (GC bias curve) is consistent, the exact structure differs remarkably across samples. Several methods have been proposed to remove GC bias, the most popular of which is the Loess regression method [41, 43, 44].

In addition, because of short length of reads and the existence of repetitive regions within the reference genome, a huge number of NGS reads cannot be clearly mapped to the reference genome. Especially for WES data, some regions of the genome have low or no coverage. Sequencing errors and mutations can lead to incorrectly mapped reads as well. These errors introduce a challenge to the alignment process resulting in a mappability bias [15]. To reduce mappability biases, CNV detection approaches typically utilize the number of uniquely mapped short reads in a sample and a normal reference and apply a Loess regression method [45, 46]. To compensate for GC and mappability biases, many RD-based methods [26, 33, 34, 44] use the ratio of sample readcounts to normal reference readcounts.

Most of the CNV detection tools focus only on reducing GC and mappability biases, which do not represent all types of possible noise, and thus considerable amount of noise remains untouched after normalization. A few CNV detection tools employ denoising techniques such as Bayesian approaches [28] and the discrete wavelet transform (DWT) [47] to reduce noise from readcount data. Signal processing techniques have been widely used for effective noise reduction. These techniques are broadly utilized to improve signal-to-noise ratio (SNR) in engineering where signals are a mixture of the original signals and various types of complex noise. However, they have had a very limited application in genomics [48–50]. Readcount data can be seen as a noisy signal with some characteristics. First, it is sparse, that means the total length of CNVs is much less that the total length of genome. Second, since copy numbers are discrete values, it is piecewise constant signal. Due to the importance of the breakpoints of CNVs, denoising methods that can preserve edges need to be used. Also, a very challenging issue in CNV identification is the difficulty of detecting focal (narrow aberration) CNV regions in the presence of extreme noise.

Because of the characteristics of readcount data and the need for the accurate detection of breakpoints and focal CNVs, in this work we used a total variation approach for denoising. In signal processing, total variation [51, 52] approaches have been very successful in removing noise from a noisy sparse piecewise constant signal while preserving edges. A noisy signal contains many unwanted details that lead to high total variation that is the summation of the absolute gradient of the noisy signal, while has few breakpoints. Therefore, a close match to the original signal can be estimated by minimizing the total variation of the signal. This optimization approach can remove unnecessary details of the noisy signal and at the same time preserve important ones such as breakpoints and narrow changes. A very efficient implementation of total variation denoising is Taut String [53], which solves the optimization problem in a non-iterative in-place manner.

The main goal of this study is to develop an efficient and effective denoising algorithm to remove biases and noise from readcount data for better identification of CNVs using NGS data. In this work, we introduced an efficient and accurate denoising technique based on a signal processing approach, Taut String [53–55]. This approach efficiently removes noise while preserves breakpoints and prepares error free readcount data for the segmentation.

## Methods

In this study, we use sparse and piecewise constant characteristics of CNV signal to reduce readcount data noise. We developed a denoising algorithm based on the Taut String approach. Before applying denoising we first filtered low quality readcount data and removed GC and mappability bias from readcount data [56]. After applying denoising, a segmentation method was used to call CNV regions from denoised readcount data. We applied the circular binary segmentation (CBS) algorithm [57] for segmentation.

### Filtering low quality readcounts

We applied a sliding window approach to compute the GC% and readcount value for each genomic window with an optional size [58]. In this work, the size of windows is 100 bp. We considered windows with readcounts and GC content in the bottom and top 1% percentiles as outliers and removed them.

### Reducing bias

Several methods have been proposed for modeling and removing GC and mappability biases from data [43, 45, 59, 60]. In order to remove GC bias, we followed the weighted Loess regression method proposed in [43]. In this method, a local weighted regression is applied to the means of the number of reads mapped to windows with a GC content of *gc* (percentage of G and C bases for each window), *m*_*gc*_s [43]. It is observed that if there are a few windows with a GC content of *gc*, then their corresponding *m*_*gc*_ values would be significantly higher or lower than *m*_*gc*_ values corresponding to other windows. The weighted Loess regression method tries to remove these local extremes, resulting in smoother values of *m*_*gc*_. Then, using the smoothed *m*_*gc*_s, the number of reads for each window will be corrected.

After applying GC bias correction on both sample and normal readcount data, we compute the ratio of sample to normal readcount for each window. These ratios can also help to mitigate mappability bias.

### Reducing noise using taut string

The accuracy of CNVs detection is heavily influenced by the noisiness of the readcount data that can be considered as readcount signals. It is observed that under highly noisy readcount signals, CNV detection tools identify many false CNVs (false positives (FPs) and false negatives (FNs)). Therefore, reducing noise is an essential step in a CNV detection algorithm.

The log2 ratios of sample and normal readcounts can be modeled as Eq. (1):

1 $$ \mathbf{r}=\mathbf{f}+\boldsymbol{\upvarepsilon} \kern0.6em , $$

where **ε** indicates noise and is defined as a vector of independent and identically distributed (iid) random variables with a normal distribution *N*(0, *σ*_*N*_^2^) (mean of 0 and standard deviation of *σ*_*N*_). A denoising method tries to recover the original signal **f** from the noisy observed signal **r**.

There are several approaches for removing noise from noisy signal. The characteristics of the noise and signal should be considered for developing an appropriate noise cancelation method. Fourier based filtering techniques [61] and Kernel estimators [62] are identified as two popular approaches for removing noise. However, when the noise and signal Fourier spectra overlap, these methods cannot separate spectra completely and fail to detect original signal [63]. Identifying small CNV segments is another challenge in a noisy environment. Usually, linear denoising approaches cannot perform well in detecting small CNV segments in low SNR environments. For a noisy readcount signal, amplitude distortion happens more often than spectra location distortion. In this situation, non-linear approaches that consider amplitudes rather than locations of the spectra in their noise cancelation procedure perform better. Furthermore, accurate detection of breakpoints plays an important role in preserving narrow CNVs while removing noise.

In this study, for estimating **f** from given noisy readcount data/signal, we employed an effective and efficient non-linear noise cancelation approach based on the total variation denoising for one-dimensional (1-D) discrete signals [53, 54] that can preserve edges and narrow segments. The total variation denoising has the ability to identify local extreme values in data with low SNR by estimating a piecewise constant signal [64, 65].

Given a noisy signal **r =** (*r*_1_, *r*_2_, …, *r*_*n*_)***,*** the goal is to estimate the denoised signal $\widehat{\mathbf{f}}$ which minimizes the eq. (2).

2 $$ \underset{f}{\min}\frac{1}{2}\ \sum \limits_{i=1}^n{\left|{r}_i-{f}_i\right|}^2+\lambda \sum \limits_{i=1}^{n-1}\left|{f}_{i+1}-{f}_i\right|\kern1em , $$

for some regularization parameter *λ* ≥ 0. The first term is used to measure the fitting error between noisy signal *r*_*i*_ and denoised signal *f*_*i*_, and the second term is used to measure the penalty caused by the difference between change-points *f*_*i*_ and *f*_*i* + 1_ using a sparsity-inducing regularizer (*λ*). The challenging part is selecting appropriate value for *λ*. In [54], *λ* is chosen as$\lambda =c{n}^{\frac{1}{2}}\sigma$ for some *C* > 0 , where  *σ* is computed as *σ* = 1.48/ √ 2 {| *r*_2_ − *r*_1_ | , …, | *r*_*n*_ − *r*_*n* − 1_ | }.

This optimization problem can generate a piecewise constant signal whose number of breakpoints, *k*, is a non-increasing function of *λ* [64]. It considers the smallest integer *k* and tries to find $\widehat{\mathbf{f}}$ with *k* local extreme values.

To solve this total variation optimization problem, we used the efficient Taut String approach. Taut String is an in-place non-iterative linear time method for 1-D TV denoising. Taut String defines a vector of running sums${R}_i=\sum \limits_{u=1}^i{r}_u$, 1 ≤ *i*≤n. Then equation (2) is converted to equation (3) using *f*_*i*_ = *s*_*i*_ − *s*_*i* − 1_ for 1 ≤ *i* ≤ *n* [66].

3 $$ {\displaystyle \begin{array}{c}\underset{s\in {\mathrm{\mathbb{R}}}^{n+1}}{\min }{\sum}_{i=1}^n\sqrt{1+{\left|{s}_i-{s}_{i-1}\right|}^2}\mathrm{subject}\kern0.17em \mathrm{to}\\ {}{s}_0=0,{s}_n={R}_n, and\ \underset{1\le i\le n}{\max}\left|{s}_i-{R}_i\right|\le \lambda \end{array}} $$

To minimize equation (3), suppose a tube of radius *ϑ* = 0.5*λ* consists of the lower bound *l*_*i*_ ≔ *R*_*i*_ − *ϑ* and the upper bound *u*_*i*_ ≔ *R*_*i*_ + *ϑ*. Then, assume that there exist a string connecting (1,*R*_1_) and (n,*R*_*n*_), restrained to lie within the tube, and it is pulled to the point that it is tight, touching the tube (at possibly multiple “knots”) on either side. Taut String tries to solve this problem by using the greatest convex minorant and least concave majorant of the upper and lower strings *R*_*i*_ + *ϑ* and *R*_*i*_ − *ϑ*. The solution of this optimization, $\widehat{\mathbf{f}}$**,** can be considered as a string between *R*_*i*_ − *ϑ* and *R*_*i*_ + *ϑ* that is pulled tight.

To improve the convergence rate at local extremes, we used the method introduced by Davies and Kovac [54] that combines the Taut String with a multiresolution bound over estimated residuals and utilizes an additional local squeezing step to the Taut String estimate. In this approach, Taut String checks if the deviation between the observed data and $\widehat{\mathbf{f}}$ satisfy the multiresolution criterion [54], if not, it uses local squeezing of tube. It means that it squeezes the tube by multiply a value *γ* ∈ (0, 1) to *λ* and obtains new upper and lower bound. This approach starts from the fixed point *s*_0_ = 0. It gradually calculates the greatest convex minorant of the upper bounds, and the smallest concave majorant of the lower bounds on the tube. A segment of the Taut String can be detected when both curves intersect. Then, the algorithm is restarted at the end of the detecting segment and is run until all Taut String segments have been identified. By employing this approach, computing $\widehat{\mathbf{f}}$is an efficient procedure and it is linear in time (*O*(*n*)) [54]. This algorithm yields piecewise constant functions. On each constant interval, the denoised values is equivalent to the mean of the corresponding observations, except for local extrema of the fit. This algorithm removes very low-frequency noise while keeping the location of change-points (breakpoints).

## Data sets

### Simulated readcount data sets

We generated simulated data to evaluate the performance of our proposed denoising method in identifying true CNVs and their breakpoints. For this purpose, 50 simulated readcount signals were generated with known true CNV segments as gold standard. Each data point represents a readcount value of a genomic window. By adding different levels of Gaussian white noise to these readcount data, we generated noisy readcount signals with different SNRs, where SNR is defined as *SNR* = (*P* _ *original signal*)/(*P* _ *noise* ). A Gaussian white noise has a perfectly flat power with ${P}_{noise}={\sigma}_N^2$.

### Simulated sequencing data sets

We used simulated WES data sets with known true CNVs as gold standard. The simulated data were generated by CNV-Sim tool (https://github.com/NabaviLab/CNV-Sim). We generated 10 data sets using CNV-Sim for chromosome 1. We aligned simulated short reads to the reference genome (hg19) using the BWA software tool [67]. We generated readcounts from aligned sequencing data by utilizing the bedtools suit [58] and employing100bp sliding window. We used these simulated data to investigate the performance of the CNV detection tools in terms of sensitivity, false discovery rate, and specificity.

### Real data sets

For this work, we downloaded 10 Breast cancer tumor and matched normal WES data from the Cancer Genome Hub (https://cghub.ucsc.edu/index.html). We used BWA to align these sequencing data. The bedtools suit [58] and 100 bp sliding window were utilized to generate readcount signals from the aligned sequencing data. The array-based CNV results from the same samples provided by the cancer genome atlas (TCGA) were used for benchmarking and evaluating the performance of the denoising methods in terms of sensitivity, false discovery rate, and specificity. We downloaded the CNV results from the SNP array platform from the Genomic Data Commons data portal (https://portal.gdc.cancer.gov).

## Results

### Simulated readcount data

**Fig. 1:**
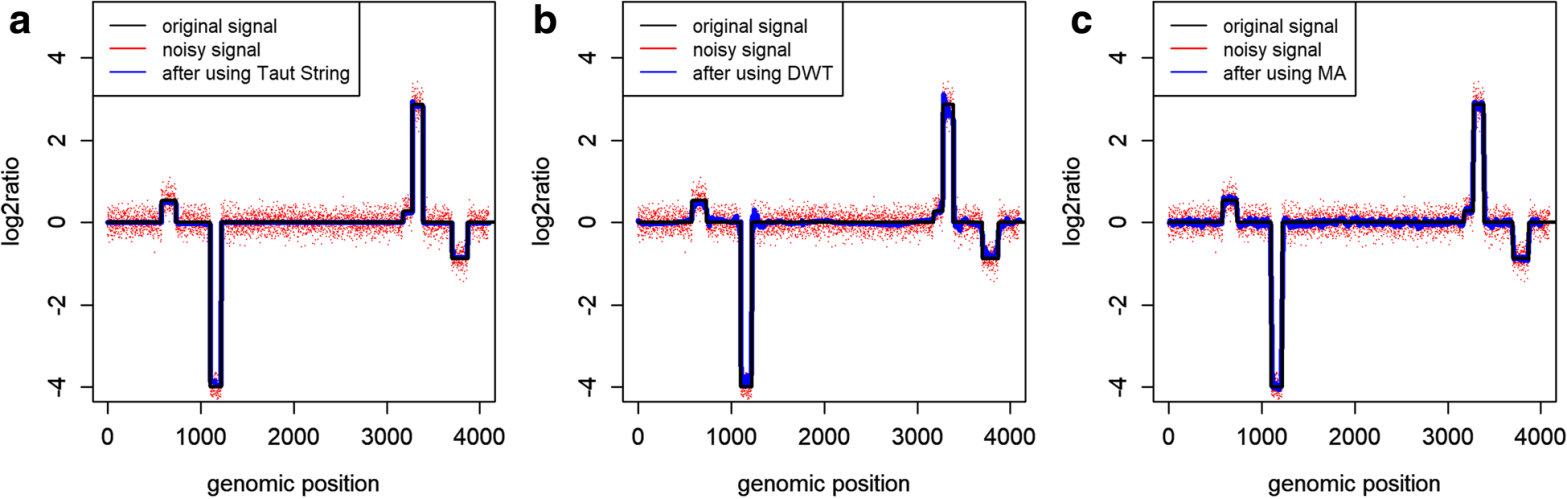
Denoising with a) Taut String and b) DWT c) MA. Using simulated readcount data at SNR = 7

In this section, we compared the performance of Taut String, with DWT and moving average (MA) denoising approaches. Figure 1 shows the simulated readcount signal before and after applying denoising methods on a noisy signal with SNR = 7. It can be seen that Taut String works better compared to DWT and MA in preserving edges and generating piecewise constant data. To compare the performance of these denoising methods in more detail, we computed sensitivities and false discovery rates (FDRs) of calling CNV segments applying the CBS segmentation. Also, we computed the breakpoint accuracy of the detected CNV segments using the denoising methods at different levels of noise. We investigated the effect of Taut String in calling true CNVs with different lengths. Furthermore, we investigated the detection power of true CNV using the Taut String denoising with different values of local squeezing *γ*.

#### The effect of the squeezing factor *γ* on the taut string performance

**Fig. 2:**
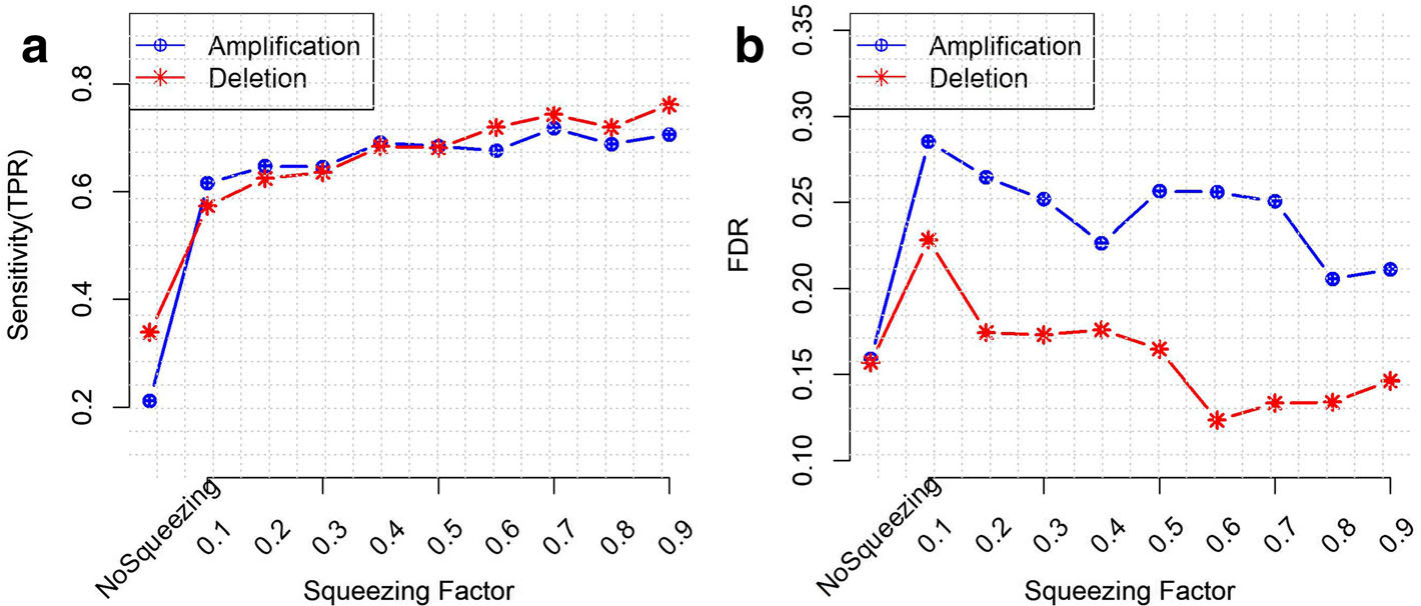
The effect of the squeezing factor on a) Sensitivity b) FDR of CNVs detection. Using simulated readcount data

We evaluated the performance of Taut String in terms of sensitivity in detecting true CNVs using different values of the squeezing factor *γ* .We used the 50 simulated readcount data sets that we explained above. Figure 2 shows that the sensitivities in calling CNV segments improve by using squeezing factors close to 1. We can see that selecting an appropriate squeezing factor is important. When *γ* is small the algorithm ends rapidly as all multiresolution coefficients satisfy the multiresolution criterion quickly and generate many local extreme values. Some of them are outlier and lead to detect more FP CNVs. When *γ* is nearly 1, the algorithm takes more time and identifies smaller number of local extreme values. Applying Taut String without using the squeezing factor results in lower sensitivity and lower FDR compared to using the squeezing factor. Considering a tradeoff between time cost and accurate detection of CNVs segments, we chose *γ* = 0.5 for the rest of our work.

#### Sensitivity and FDR of identifying CNV segments

Using the 50 sets of simulated readcount signals with different levels of noise (*σ*_*N*_ from 0.47 to 0.05), we compared the sensitivities in detecting CNV segments using DWT, Taut String and MA. Segment-based comparison [18], which considers the overlap between detected CNVs regions and benchmark CNVs, was used to compute true positives (TPs), FN, and FPs. The segment-based FPs and TPs were used to calculate sensitivities, specificities and FDRs. “GenomicRanges” R package from Bioconductor [68] is applied to calculate overlapping regions between detected CNVs and benchmark CNVs. A threshold of ± *thr* for *log*_*2*_*ratios* was used for calling CNV segments. TP happens when a detected amplified/deleted segment has an overlap of 80% or more with a benchmark amplified/deleted segment. FN happens when an amplified/deleted segment in the benchmark does not have an overlap of 80% or more with any detected amplified/deleted regions. FP happens if there is no overlap of 80% or more between a detected CNV region and any benchmark CNVs. Sensitivity and FDR are defined as *TP*/(*FN* + *TP*) and *FP*/(*FP* + *TP*), respectively.

**Fig. 3:**
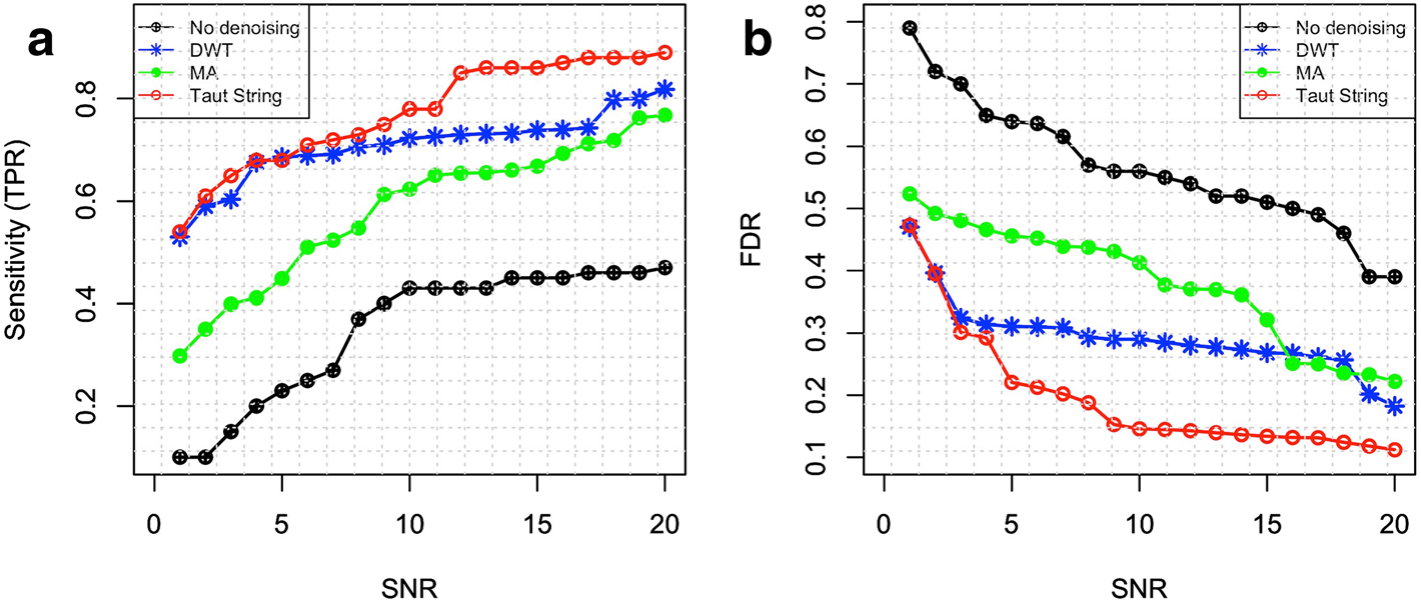
a) Sensitivity and b) FDR of detection of amplified CNVs segments before and after applying denoising methods for different *SNR*. Using Simulated Readcount data

**Fig. 4:**
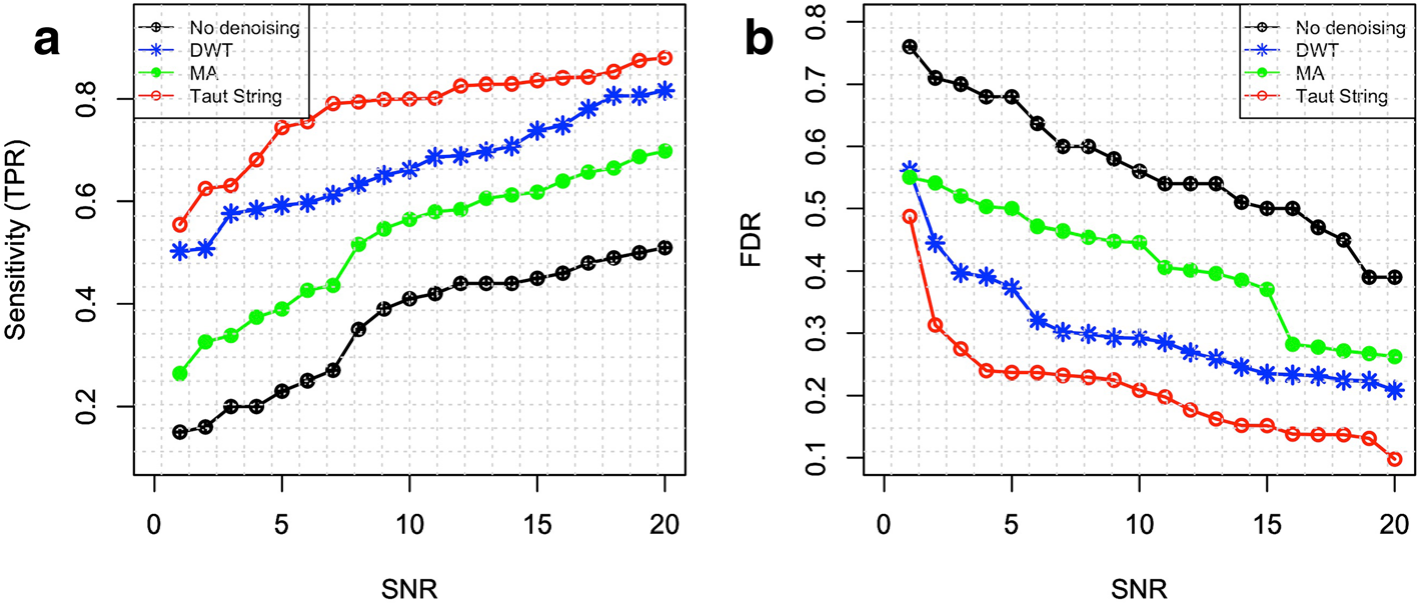
a) Sensitivity and b) FDR of detection of deleted CNVs segments before and after applying denoising methods for different *SNR*. Using simulated Readcount data

Figures 3 and 4 show the sensitivity and FDR of detecting amplified and deleted segments, respectively, with *thr* = 0.2. It can be seen that all three denoising methods improve sensitivity and FDR of CNV detection. However, edge protecting approaches (Taut String and DWT) can improve sensitivity and FDR significantly better than MA, and Taut String outperforms DWT in detecting CNVs. All three methods perform better for higher SNRs. Even at a high SNR condition, the segmentation algorithm without denoising provides so many false detections, where employing Taut String can result in near perfect detection.

#### Breakpoint accuracy in different level of noise

**Fig. 5:**
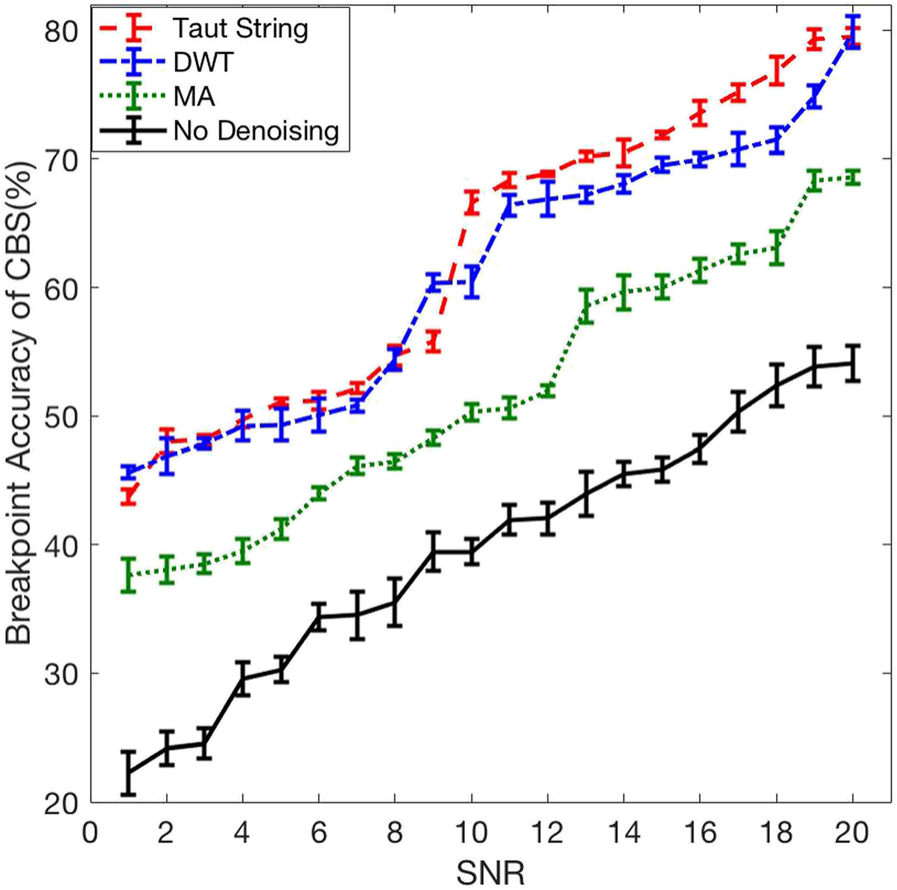
Breakpoint accuracy before and after applying denoising for different SNR, using simulated readcount data

The three denoising methods DWT, MA and Taut String were applied to the 50 simulated readcount data with different level of SNRs ranging from 1 to 20 (*σ*_*N*_ from 0.47 to 0.05). Then, CNVs’ segments were identified from the denoised and noisy readcount data by using CBS. We defined breakpoint accuracy as the frequency of identifying exactly true start and end points of detected CNVs’ segments. We used the known start and end points of simulated CNVs’ segments to compute these frequencies. Figure 5 shows the performance of DWT, Taut String and MA in detecting breakpoints. As also depicted in Fig. 1, we can observe that using an appropriate smoothing approach before segmentation improves the breakpoint accuracy significantly. It can be seen that DWT and Taut String outperform MA. At lower levels of SNR (higher noise), DWT and Taut String perform almost similar but at higher levels of SNRs Taut String performs slightly better than DWT. For having a high accuracy of CNV breakpoints detection, the denoising method should provide sharp edges with less fluctuation around the edges. Denoised signals using Taut String show less fluctuation at the breakpoints compared to DWT and MA (Fig. 1). Taut String denoising is more powerful to preserve edges leading to better performance in breakpoint detection.

#### The effect of denoising methods in detecting CNVs with different lengths

**Fig. 6:**
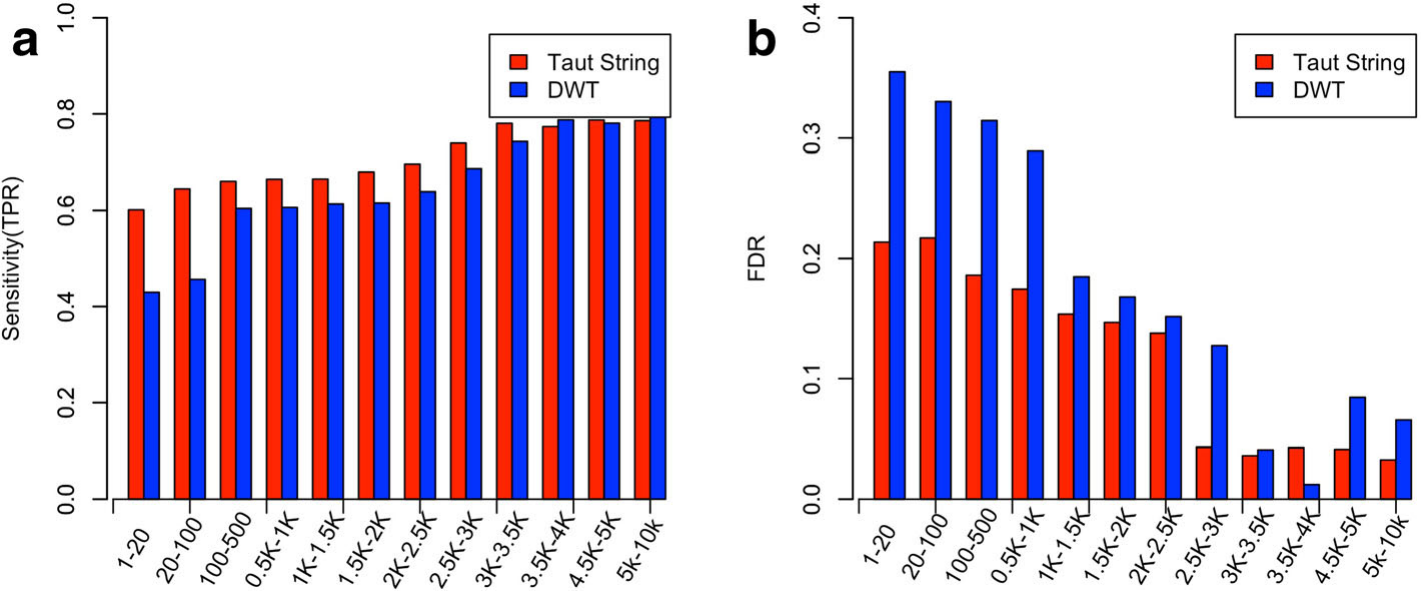
Sensitivity and FDR of detection of amplified CNV segments with different CNV lengths

**Fig. 7:**
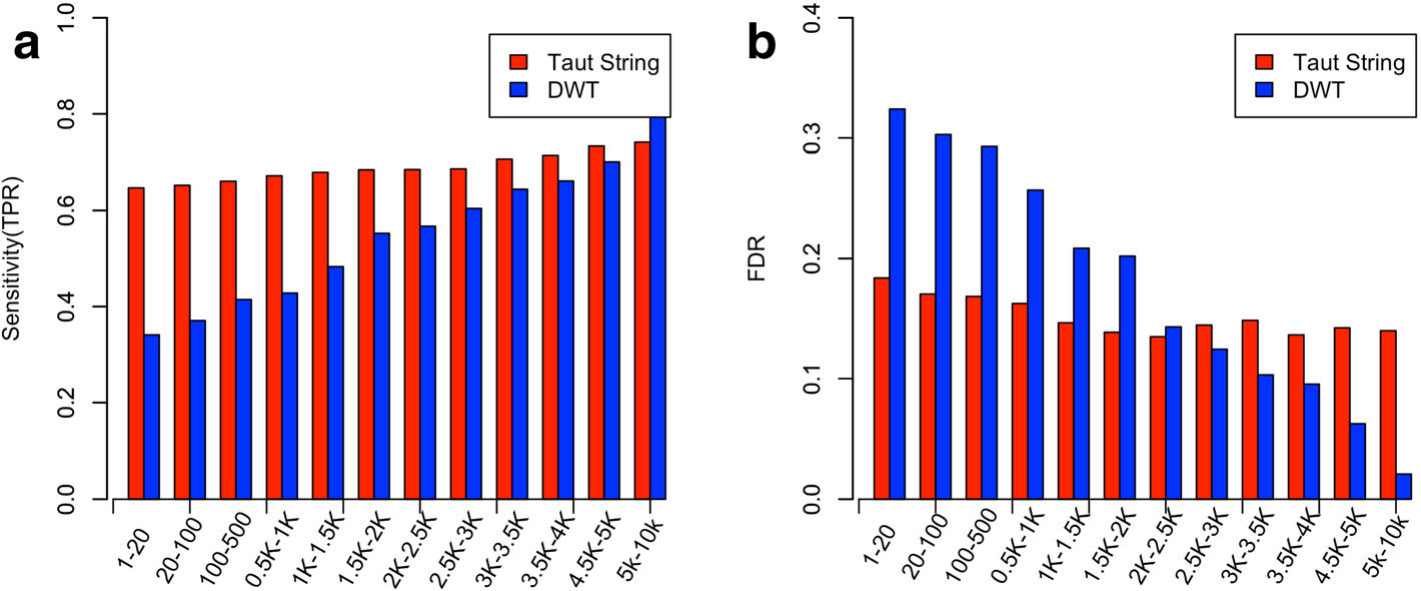
Sensitivity and FDR of detection of deleted CNV segments with different CNV lengths

Each data point in simulated data represents a readcount value of a sliding window. Simulated data sets contain CNV segments with different length (in window size) ranging from 1 to 10 k. Figures 6 and 7 show the sensitivities and FDRs in detecting CNVs with different length using Taut String and DWT. We can see that in general sensitivity and FDR is better in detecting larger CNVs. However Taut String outperforms DWT for narrower CNVs. For CNV segments with lengths between 1 and 20, sensitivity in detecting amplification/deletion is 0.60/0.66 when using Taut String, and is 0.42/0.34 when using DWT. We can see a stronger pattern in FDRs. For CNV segments with lengths between 1 and 20, FDR in detecting amplification/deletion is 0.21/0.18 when using Taut String and is 0.35/0.32 when using DWT.

### Simulated sequencing data using CNV-Sim

As explained above, we generated 10 simulated paired-end WES data sets with read length of 100 bp for chromosome 1 using the CNV-Sim simulator. Using simulated sequencing reads from genomes that contain known CNVs, we calculated sensitivities and FDRs of detecting CNVs with and without using denoising. We used the lists of known simulated CNV for benchmarking.

To calculate Sensitivity and FDR, we used a gene-based approach [18]. First, we used CBS to call CNV segments and then we annotated the identified CNV segments to derive CNV gene lists. We used “cghMCR” R package from Bioconductor [69] to identify CNV genes using Refseq gene identifications. A threshold of ±*thr* for *log*2 *ratios* was used to detect CNV genes, that is: amplification for *log*2 *ratios* > *thr*, deletion for *log*2 *ratios* <  − *thr*, and No CNV for *log*2 *ratios* between −*thr* and *thr*. Here, we used *thr* = 0.5. Table 1 shows definitions for sensitivity (true positive rate (TPR)), FDR or 1-Precision, and specificity (SPC). Table 2 shows overall performance of the denoising methods using the simulated short read data.

**Table 1:** Possible results for each candidate CNV genes

CNV gene	Not identified	Identified
Present	FN	TP
Not present	TN	FP
Performance metrics:		
Sensitivity =$\frac{TP}{FN+ TP}$	FDR =$\frac{FP}{FP+ TP}$	Specificity = $\frac{TN}{FP+ TN}$

**Table 2:** Overall Performance of The Denoising Methods Using the simulated WES data generated by CNV-Sim Data

Denoising Methods	Amplified CNVs			Deleted CNVs		
	Sensitivity	FDR	Specificity	Sensitivity	FDR	Specificity
Before applying denoising method	79.65%	35.23%	80.93%	78.64%	37.03%	81.02%
After applying DWT	86.87%	22.88%	91.32%	87.20%	20.54%	90.32%
After applying Taut String	87.17%	22.94%	92.82%	88.15%	23.65%	89.49%

Similar to simulated readcount data we observed that denoising improves the performance of CNV identification; and Taut String outperforms DWT. Using the Taut String denoising, the sensitivity of detecting amplifications improves from 79.65 to 87.17% and the sensitivity of detecting deletions improves from 78.64 to 88.15%.

### Real data

To investigate the performance of the denoising methods in identifying CNVs, we evaluated the results of CNV detection with and without applying denoising methods using the real data sets. The results are shown in Table 3. It can be seen that denoising methods improve the performance of the CNV detection. In overall, by using the Taut String denoising method, the sensitivity of detecting amplifications improves from 50.99 to 69.52% and the sensitivity of detecting deletions improves from 60.37 to 79.93%. As expected, Taut String works better than DWT in denoising real readcount data results in higher sensitivity and specificity in CNV detection. Removing noise by Taut String and DWT, increases the number of both TP and TN and decreases the number of both FP and FN leading to improving the overall performance of a CNV detection method.

**Table 3:** Overall Performance of The Denoising Methods Using the Real WES Data

Denoising Methods	Amplified CNVs			Deleted CNVs		
	Sensitivity	FDR	Specificity	Sensitivity	FDR	Specificity
Before applying denoising method	50.99%	42.06%	80.45%	60.37%	64.32%	56.71%
After applying DWT	68.81%	41.65%	79.92%	77.65%	54.32%	72.23%
After applying Taut String	69.52%	40.21%	84.51%	79.93%	50.72%	77.25%

Using the real data sets, we compared the copy number values, in log2 ratios, of detected CNV genes with those of their corresponding benchmark CNV genes, before and after applying Taut String and DWT. Results show that after using Taut String, copy number values of 76.87% of amplified genes and 70.26% of deleted genes differ from their benchmark copy number values less than 20% of the benchmark copy number values. Table 4 shows the results when using no denoising, DWT and Taut String.

**Table 4:** Percentage of CNV genes that the difference between their copy number values from their benchmark values are less than 20% of the benchmark copy number values

Denoising methods	Amplified genes	Deleted genes
no denoising	44.48%	42.77%
DWT	73.36%	58.35%
Taut String	76.87%	70.26%

When using Taut String denoising, the average of differences between the detected copy number values and the benchmark copy number values are %25 and %36 of the benchmark copy number values across all amplified and deleted genes, respectively. These averages when using no denoising and using DWT are shown in Table 5. Taut String outperforms DWT in providing more accurate copy number values as shown in Table 4 and Table 5. As can be seen in these tables, using denoising is beneficial in terms of improving accuracy of copy number values as well.

**Table 5:** Average differences between detected copy number values and benchmark copy number values respect to the benchmark copy number values

Denoising methods	Amplified genes	Deleted genes
no denoising	50%	53%
DWT	38%	36%
Taut String	25%	36%

### Runtime comparison

CBS segmentation is the most commonly used and effective segmentation methods; however, it is slow when readcount data are very noisy. This is because it uses an iterative algorithm based on the variance of the data. Denoising methods that smooths readcount data can help to speed up the segmentation by CBS. As we show in Fig. 1, Taut String generates smoother data with more clear edges (low fluctuations at breakpoints), which results in faster segmentation by CBS. In this section, we calculated the overall runtime of denoising and segmentation algorithm together using real and simulated data sets on a 64-bit Windows 10 Operating System, with intel core i7-7500 U 2.7 GHz CPU and 16 GB DDR4 memory. The time complexity of Taut String and DWT are *O*(*n*) and *O*(*nlogn*) respectively [70]. From the runtime perspective, applying Taut String and CBS algorithms subsequently surpasses applying DWT and CBS. Using the real data sets, on average, DWT and CBS combination took 76.35 min while Taut String and CBS combination took only 21.23 min. We observed similar behavior using simulated data. On average, DWT and CBS took 30.67 s while Taut String and CBS took only 10.45 s.

## Discussion

Readcount data’s noise and biases distort the association between copy numbers and read coverages. These biases and noise need to be removed from noisy readcount data in order to have more accurate CNV identification. In this study, we proposed to use a signal processing approach based on total variations and Taut String to reduce readcount noise. In general, denoising improves sensitivity and FDR of CNV detection. However, edge protecting denoising approaches (e.g. Taut String and DWT) significantly outperform regular denoising methods (e.g. MA). In fact, a denoising method that can generate less fluctuations and sharper edges at breakpoints, such as Taut String, leads to detecting more accurate CNVs compared to other methods (e.g. DWT and MA). Using simulated and real data, we showed that Taut String outperforms DWT and MA approaches in terms of sensitivity, FDR, and specificity in CNV detection. The major advantage of Taut String is its ability in preserving CNV segments’ breakpoints, resulting in increasing the detection accuracy of a CNV segmentation method, especially in detecting narrow CNVs. Due to Taut String power in estimating piecewise constant signals, the identified CNV segments have more accurate breakpoints and copy number values. In addition, the Taut String method is very efficient. The complexity of its algorithm is linear in time. NGS data are big and using an efficient and fast CNV detection method is essential. The proposed method is an effective and practical approach to improve CNV identification due to its high efficiency and its power to detect true CNVs. However, adjusting the optimization parameter for Taut String, which indicates the upper bound and lower bound of error is challenging. In order to have a high accurate denoising approach, selecting an appropriate error bound is critical. Having a global error bond reduces the effectiveness of Taut String. Local squeezing that reduces the error bond locally by a constant factor improves the performance of Taut String. When this constant factor is small, the algorithm takes less time and many extreme values will be generated. In contrast, when this constant factor is close to one, we can have more accurate CNV detection while the computational time increases.

## Conclusions

Denoising readcount data for CNV detection methods that are based on depth of coverage, can remarkably improve the accuracy of CNV detection. However, most of the current CNV detection tools do not employ denoising techniques, which results in low sensitivity and high false positive rates. Also, noise cancellation algorithms need to be very efficient in order to not increase the overall complexity of CNV identification. In this work, we developed an efficient and effective denoising method based on signal processing approaches. The proposed method uses the total variation approach for cancelling noise and employs the non-iterative, in-place Taut String algorithm to obtain the optimal approximation of denoised data. Signal processing approaches have a long history in noise cancellation and can be extremely valuable for improving the accuracy of CNV detection. Selecting an appropriate denoising approach depends on the characteristics of the noise and signal. From a signal processing point of view, readcount data are sparse, discrete, and piecewise constant. Noise cancelation algorithms for these types of signals usually are Fourier based denoising methods and Kernel estimators. These techniques cannot separate spectra correctly. As a result, they can reduce noise, but are not able to preserve edges. By contrast, total variation denoising methods can simultaneously preserve edges, remove noise, and generate piecewise constant signals, even at high levels of noise. We used Taut String for efficient implementation of total variation denoising. To investigate the performance of the Taut String denoising approach, we compared the accuracy of detecting CNV segments when using Taut String denoising with those of when using DWT and MA denoising methods. DWT, which is commonly used in bioinformatics, is also an efficient and nonlinear smoothing method. However, this study showed that Taut String outperform DWT in both efficiency and accuracy.

## Abbreviations

CBS: Circular Binary Segmentation
CNV: Copy Number Variation
DWT: Discrete Wavelet Transform
FDR: False Discovery Rate
FN: False Negative
FP: False Positive
FPR: False Positive Rate
MA: Moving Average
SPC: Specificity
TCGA: The Cancer Genome Atlas
TN: True Negative
TP: True Positive
TPR: True Positive Rate
WES: Whole Exome Sequencing
WGS: Whole genome Sequencing
